# Cemento-Ossifying Fibroma of the Craniofacial Region: A Diagnostically Challenging Case Report

**DOI:** 10.7759/cureus.94539

**Published:** 2025-10-14

**Authors:** Jay M Patel, Samir Joshi, Sudhir Pawar, Vivek Nair, Vaishali Koranne

**Affiliations:** 1 Oral and Maxillofacial Surgery, Bharati Vidyapeeth (Deemed to be University) Dental College and Hospital, Pune, IND; 2 Oral Medicine and Radiology, Bharati Vidyapeeth (Deemed to be University) Dental College and Hospital, Pune, IND

**Keywords:** benign fibro-osseous lesion, cementoma, cemento-ossifying fibroma, odontogenic tumor, periodontium

## Abstract

Cellular fibrous connective tissue, which eventually mineralizes, replaces normal bone in a class of illnesses known as benign fibro-osseous lesions. Fibrous dysplasia, ossifying fibroma, and osseous dysplasia are the three most common forms of benign fibro-osseous lesions. Because of their overlapping clinical, radiological, and histological features, many disorders can be challenging to diagnose. Cemento-ossifying fibroma (COF) is considered a central neoplasm of mesenchymal origin that differentiates into bone and the periodontium. This has also sparked significant debate over its terminology and diagnostic criteria.

In the case described, the patient presented with a painless, expansile swelling in the right mandible, which, following histopathological confirmation of COF, was surgically removed. This approach aligns with evidence indicating that total excision is the gold standard treatment for optimal outcomes.

## Introduction

A well-circumscribed radiolucent lesion that can be unilocular or multilocular and has varying degrees of radiopacity depending on the type of mineralized tissue present is the hallmark of cemento-ossifying fibroma (COF), an uncommon, benign fibro-osseous lesion found in the jaw [1]. Although it is more common in the mandible, it can occur in the maxilla as well. The tumor is made up of highly cellular fibrous tissue with varying amounts of calcified material that resembles bone, cementum, or both. The craniofacial area is the primary site of COF, which often manifests in the second or third decade of life and is more prevalent in females than males, with a 1:4 ratio [2]. Initially, the World Health Organization (WHO) classified it as a fibro-osseous tumor. The condition is marked by painless expansion of the cortical bone plates, and it can generally be treated with conservative surgical excision, with a high recurrence rate [3].

## Case presentation

**Figure 1:**
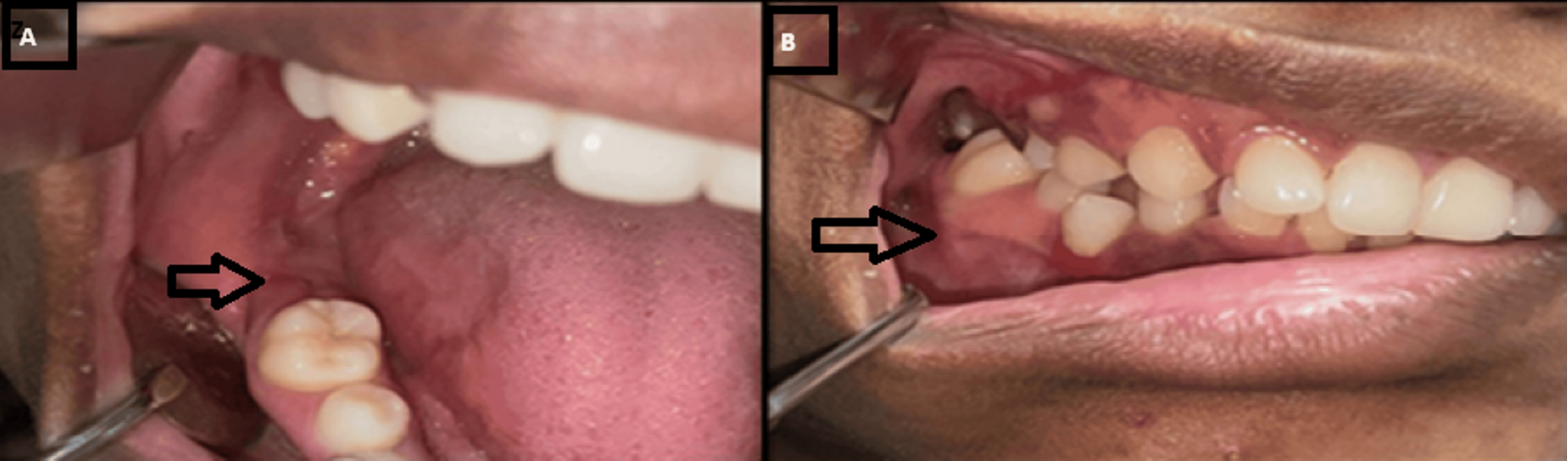
Pre-operative photograph showing the mandibular posterior region (A) Lingual cortical expansion observed in the mandibular posterior region. (B) Buccal cortical expansion observed in the mandibular posterior region.

A 25-year-old woman's dentist recommended her to the oral and maxillofacial surgery department because of swelling in an area of her right lower back tooth that had been there for two to three months. There was no relevant medical or family history, and no reported history of any trauma for the patient. There was no visible cervical adenopathy, hard swelling, or enlargement on extraoral inspection. A reddish, spherical, well-defined, firm swelling was seen in the right mandibular body area upon intraoral examination, along with buccal and lingual cortical bone growth. It was non-tender and asymptomatic (Figure 1). The lesion was expansile in nature, which resulted in severe facial asymmetry.

**Figure 2:**
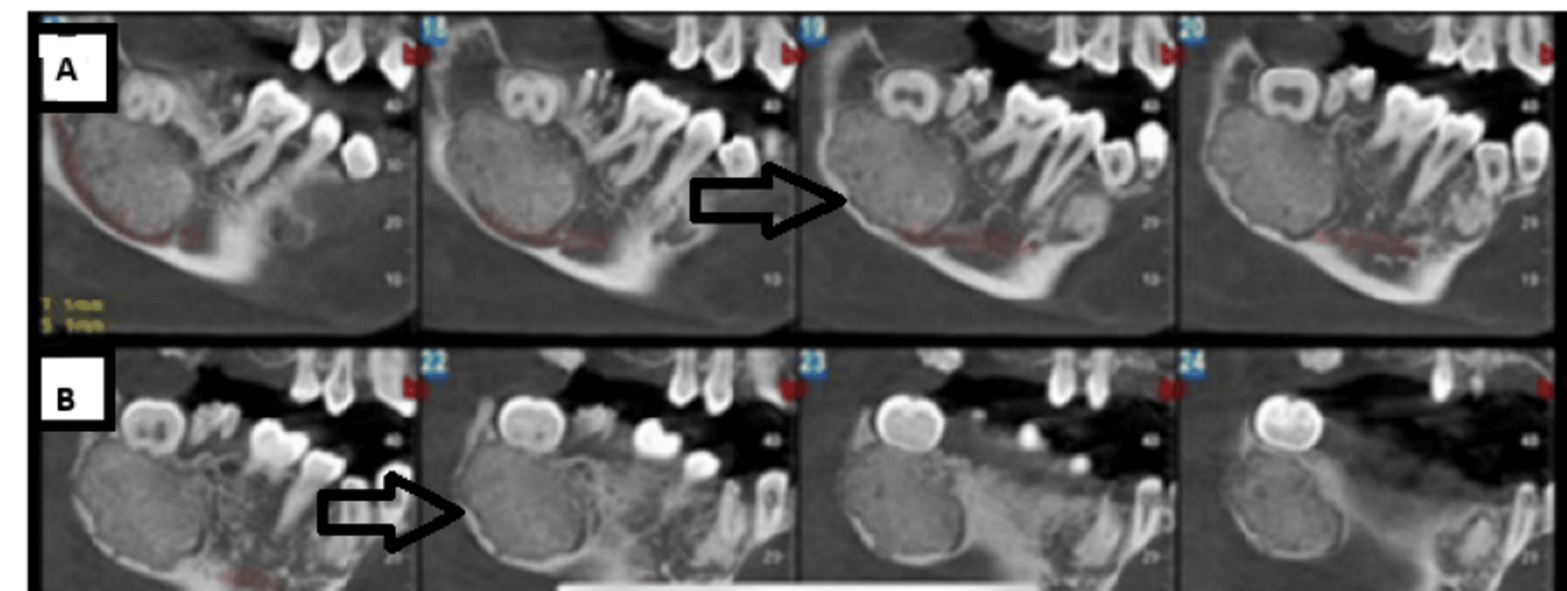
Cone-beam computed tomography (CBCT) showing well-defined and encapsulated radiopacity involving the mandibular posterior region (A) The CBCT slice shows the relation of the inferior alveolar nerve to the lesion. (B) The CBCT slice shows the extent of the lesion.

**Figure 3:**
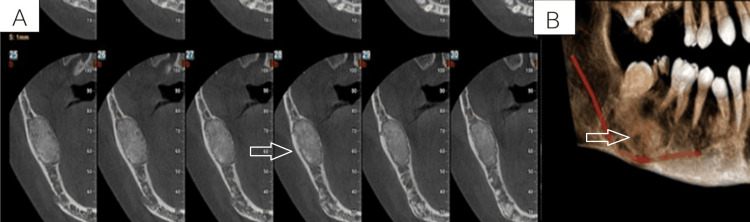
(A) Cone beam computed tomography (CBCT) showing cortical expansion; (B) 3D CBCT view showing the encapsulated lesion involving the mandibular posterior region

Recent radiographic examination reveals well-defined radiopacity in the region apical to the right third molar. The approximate dimensions of the lesion are 13.0 x 24 x 18.2 mm. A decrease in the thickness of the mandibular inferior border and an inferior displacement of the inferior alveolar nerve canal are indicative of the lingual and buccal cortical plate's thinning and expansion. The lesion is noted with a well-defined radiolucency surrounding the radiopaque mass (Figures 2-3). However, owing to the considerable overlap in radiographic features among fibro-osseous lesions, including similarities in the pattern of mineralization and the degree of cortical expansion, establishing a definitive diagnosis proved challenging.

**Figure 4:**
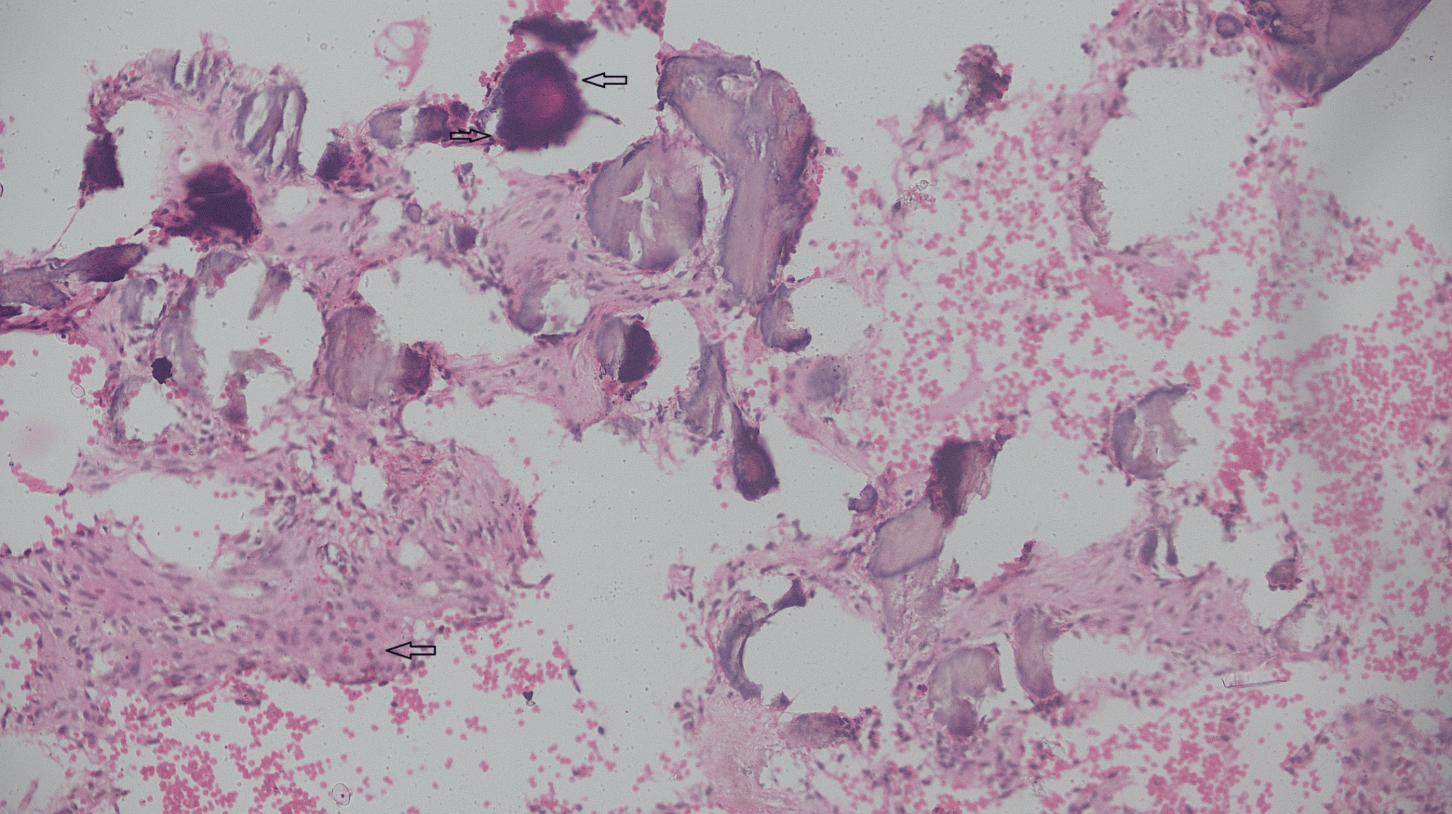
The histopathological specimen presented with fibrillar connective tissue in between fibrous connective tissue and ossicles, with the presence of abundant irregular psammomatous basophilic nodules

The biopsy performed in the oral surgery department, under local anesthesia, revealed fibrillar connective tissue interspersed with abundant plump fibroblasts. Between the fibrous connective tissue, irregular osseous trabeculae with osteoblastic rimming and numerous osteocytes in lacunae, suggestive of ossicles, with the presence of abundant irregular psammomatous basophilic nodules and calcifications suggestive of cementicles can be observed (Figure 4).

**Figure 5:**
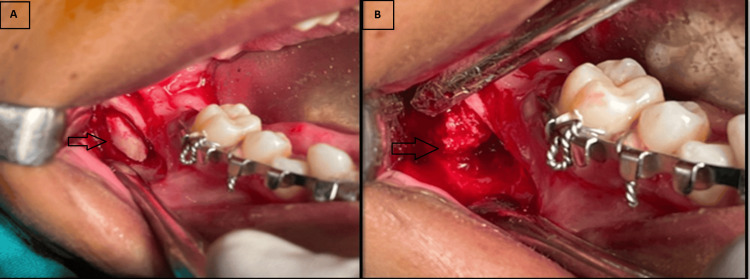
Intraoperative enucleation and curettage of the tumor were performed. (A) Bone window formation for access to the lesion. (B) Enucleation of the fibro-osseous lesion.

Surgery was planned under general anesthesia. Prior informed consent was taken primarily for enucleation of the tumor, curettage, and peripheral osteotomy because of the patient's age, the intact inferior border of the mandible, and the lack of involvement of any nearby anatomical structures (Figure 5). The excised specimen was submitted for histopathological examination, the findings of which were consistent with the preoperative biopsy report, thereby confirming the diagnosis of COF.

**Figure 6:**
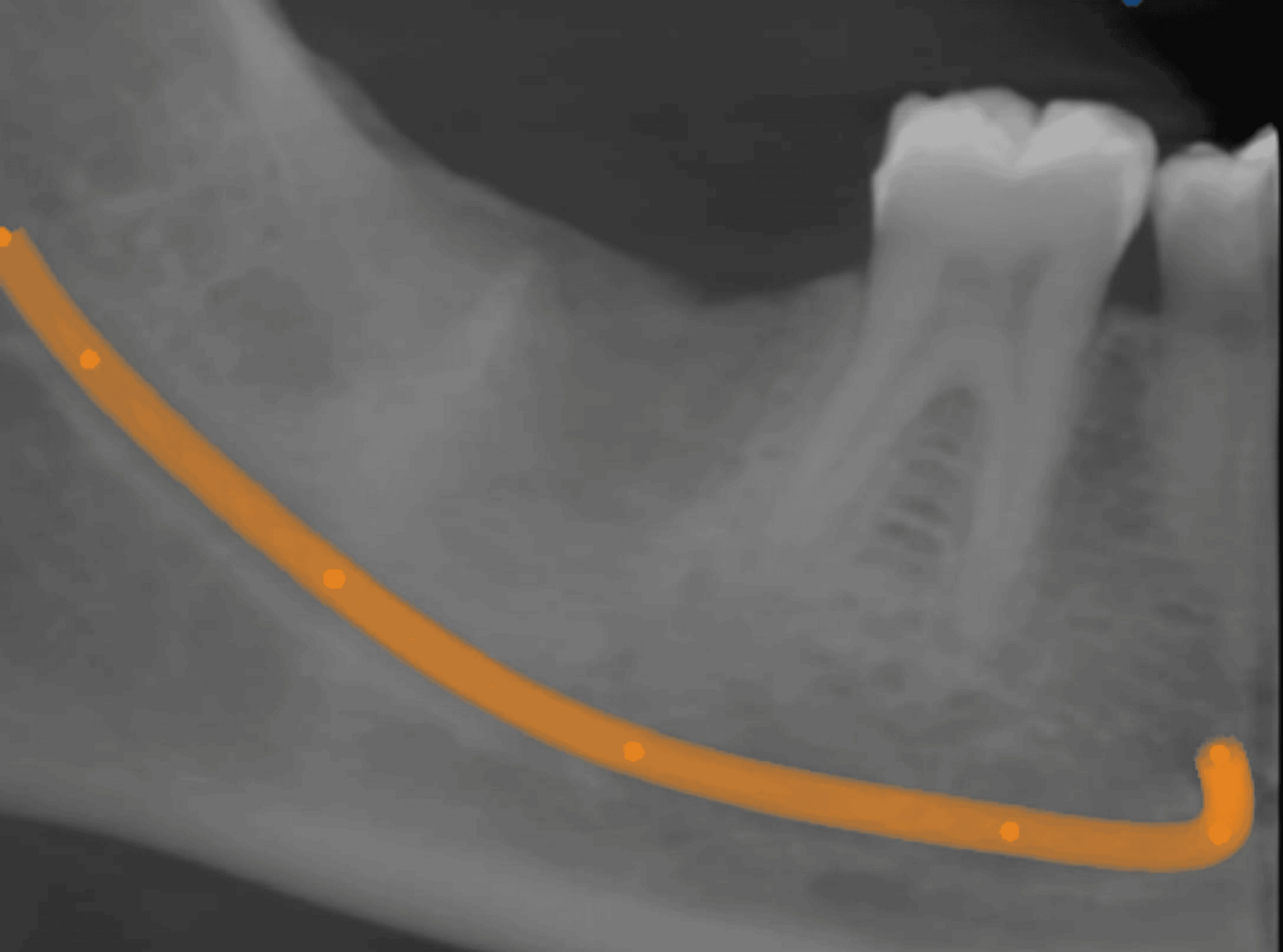
Postoperative six-month follow-up cone beam computed tomography reconstructed panoramic projections show osseous regeneration.

Postoperative follow-up was taken after six months. Clinically, no significant abnormality was detected. Cone beam computed tomography (CBCT) reconstructed panoramic projections were taken, which showed osseous regeneration and a normal growth pattern (Figure 6).

## Discussion

The most recent classification of the WHO of benign tumors of the head and neck (2017) addresses the issue regarding COF by classifying it as an odontogenic tumor of mesenchymal origin. COF is believed to originate from the mesenchymal blast cells of the periodontal ligament, which are composed of cementum, bone, and fibrous tissue. Two possible explanations for the high levels of mesenchymal cellular induction into bone and cementum that lead to odontogenesis are the emergence of a metaplastic process in the connective tissue fibers and the irregular proliferation of periodontal ligament cells [3].

Four different forms of fibro-osseous neoplasms, formerly known as cementomas, were identified by the WHO in 1992: fibrous dysplasia, OF, COFs, and cementifying fibroma [3]. Clinically, they look like a slowly expanding mass that is usually located in the mandibular premolars and molars; however, they can also arise in the orbital, temporal, nasal, and paranasal bones on occasion. They can induce symptoms like purulent rhinorrhea and orbital displacement of the neoplastic lesions when they affect the nasal or orbital bones [3]. Although benign, COFs can occasionally appear as a central variant that may lead to neoplastic changes. Juvenile aggressive ossifying fibroma is an aggressive form of COF [4]. Between the ages of 5 and 15, juvenile aggressive ossifying fibroma (JAF) is more vascular and aggressive [3]. Overall, COF is asymptomatic, well-defined, and encapsulated. However, as it grows, it may eventually cause facial deformation, necessitating surgery [5]. The only clinical characteristic of the resulting facial asymmetry could be tooth displacement. Root resorption is rare in the case of COFs, and the possible etiological factors of COFs include trauma or extractions [3]. It might be difficult to distinguish COF from calcifying odontogenic cysts and calcifying epithelial odontogenic tumors on radiographic imaging if it is associated with impacted teeth [6]. The differential diagnosis for COF radiographically includes florid cemento-osseous dysplasia or cementoblastoma if it is seen around tooth roots. When examined under a microscope, COF shows numerous delicately interlacing collagen fibers that are rarely grouped into distinct bundles, together with a significant number of active, growing cementoblasts and fibroblasts [6]. The trabeculae of lamellar or woven bone may be present alone or in combination with spherical, cementicle-like masses. COFs were found to exhibit strong immune reactivity for keratan sulfate, whereas ossifying fibromas and fibrous dysplasias showed strong immunostaining for chondroitin-4-sulfate [1,4]. One of the following methods--enucleation, curettage, or surgical excision of the tumor with or without a continuity defect--is frequently used in surgical therapy for COF [1]. The approach is primarily determined by the tumor's clinical and radiological appearance. Enucleation and curettage is required for smaller lesions, and a larger lesion would require surgical excision [3]. Because of its propensity to reappear after incomplete removal, larger COFs necessitate more drastic therapy. Resection and rebuilding with either vascularized or non-vascularized free flaps should be performed, depending on the lesion's size and volume. Because of the high incidence of radiation-induced sarcomas and the radio-resistant nature of the lesions, an ossifying fibroma cannot be treated with irradiation. Smaller lesions should be treated with enucleation and primary closure. The lesion's prognosis is generally favorable, and recurrence following tumor removal is unlikely. However, because maxillary COF is larger at presentation and more difficult to remove surgically than mandibular COF, numerous researchers have suggested that the lesion be completely surgically removed as soon as possible [1,4]. Since recurrences can happen up to 10 years after therapy, these patients must be monitored for an extended period of time; the recurrence rate is 10.1% [7]. No study justifies the malignant transformation of COFs [1].

## Conclusions

Cemento-ossifying fibroma (COF) is a rare benign fibro-osseous tumor of the maxillofacial skeleton that grows gradually and has a distinct shape. Since there are no behavioral differences between cemento-ossifying and other fibro-osseous lesions, diagnosis may be difficult. Histopathologic correlation can be helpful for a definitive diagnosis. Large lesions should be surgically excised, although well-defined lesions can be treated with enucleation and curettage. A thorough surgical treatment plan with extended follow-up is required in instances of recurrence.
